# Dose-Dependent Response to the Environmental Pollutant Dichlorodipheniletylhene (DDE) in HepG2 Cells: Focus on Cell Viability and Mitochondrial Fusion/Fission Proteins

**DOI:** 10.3390/toxics9110270

**Published:** 2021-10-20

**Authors:** Mario Alberto Burgos Aceves, Vincenzo Migliaccio, Marilena Lepretti, Gaetana Paolella, Ilaria Di Gregorio, Serena Penna, Caterina Faggio, Lillà Lionetti

**Affiliations:** 1 Department of Chemistry and Biology “A. Zambelli”, University of Salerno, Via Giovanni Paolo II, 132, 84084 Fisciano, Italy; marioburgos21@hotmail.com (M.A.B.A.); vmigliaccio@unisa.it (V.M.); mleprettil@gmail.com (M.L.); gpaolella@unisa.it (G.P.); idigregorio@unisa.it (I.D.G.); spenna@unisa.it (S.P.); 2 Department of Chemical, Biological, Pharmaceutical, and Environmental Sciences, University of Messina, Viale F. Stagno d’Alcontres, 31, 98166 Messina, Italy; cfaggio@unime.it

**Keywords:** environmental pollutants, hepatocytes, mitochondria, Mfn2, DRP1, SOD

## Abstract

Dichlorodiphenyldichloroethylene (DDE), the primary persistent metabolite of dichlorodiphenyltrichloroethane (DDT), has toxic effects on cells, but its dose-dependent impact on mitochondrial proteins involved in mitochondrial fusion and fission processes associated with cell viability impairment has not yet been analysed. Mitochondrial fusion and fission processes are critical to maintaining the mitochondrial network and allowing the cell to respond to external stressors such as environmental pollutants. Fusion processes are associated with optimizing mitochondrial function, whereas fission processes are associated with removing damaged mitochondria. We assessed the effects of different DDE doses, ranging between 0.5 and 100 µM, on cell viability and mitochondrial fusion/fission proteins in an in vitro hepatic cell model (human hepatocarcinomatous cells, HepG2); the DDE induced a decrease in cell viability in a dose-dependent manner, and its effect was enhanced in conditions of coincubation with dietary fatty acids. Fusion protein markers exhibited an inverted U-shape dose-response curve, showing the highest content in the 2.5–25 μM DDE dose range. The fission protein marker was found to increase significantly, leading to an increased fission/fusion ratio with high DDE doses. The low DDE doses elicited cell adaption by stimulating mitochondrial dynamics machinery, whereas high DDE doses induced cell viability loss associated with mitochondrial dynamics to shift toward fission. Present results are helpful to clarify the mechanisms underlying the cell fate towards survival or death in response to increasing doses of environmental pollutants.

## 1. Introduction

Mitochondria are vital organelles involved in ATP production and in regulating apoptotic and necrotic cell death [1,2,3]. Suitable mitochondrial quality should be maintained in cells to avoid metabolic alterations and cell death [4,5]. Mitochondrial toxicity is a crucial determinant in pathogenesis and/or biological adaptation to various chemical exposures [6]. They represent the primary or secondary site of action of several chemical compounds, which can induce an increase in the reactive oxygen/nitrogen species (ROS/RNS) levels in cells [1,7,8,9,10,11,12,13,14,15,16,17,18]. Therefore, an area of rapid research growth could be represented by studies analyzing the mechanisms by which different environmental chemical compounds could affect mitochondria and cellular redox homeostasis. Dichlorodiphenyl trichloroethane (DDT) and its primary metabolite, dichlorodiphenyldicloroethene (DDE), are organochlorine pesticides that act as endocrine-disrupting chemicals. They induce hormonal and metabolic disorders and impair mitochondrial bioenergetics [19,20]. DDT was primarily used to control malaria cases during the Second World War and the post-war period. Given its efficiency as a chemical insecticide, DDT utilization was massively extended to agriculture, leading to an accumulation in soil, water surface and other environmental compartments [21,22]. DDT for agriculture has been banned in different countries due to its adverse effects on wildlife and its risk for human health. However, WHO still encourages DDT use for sanitary purposes in equatorial world zones where malaria cases persist and are endemic [23]. Given its lipophilic property, human exposure to DDT is mainly associated with consuming contaminated fatty foods (such as fish, milk, and fat meat) [24]. DDT and DDE can be biomagnified in the food chain [25] and bioaccumulated into adipose tissue and liver [26,27,28]. According to the US National Cancer Institute (NCI), the liver seems to be the main target organ of DDE in mammalian species [29], probably due to the high sensitivity of hepatic cells towards xenobiotics exposure [27]. Moreover, metabolic detoxification processes mainly take place in hepatocytes [30,31] by involving the endoplasmic reticulum (ER), mitochondria, and lysosomes [32]. Mitochondrial bioenergetics injury by DDT and DDE exposure has been reported in humans and wildlife [20]. Indeed, DDT and DDE impaired the mitochondrial respiratory chain and, consequently, ATP production [20]. Moreover, impairments in protein content and enzymatical activity of cytosolic and mitochondrial antioxidant enzymes were associated with DDT/DDE exposure [17,33,34]. Mitochondrial dysfunction has been associated with an elevation in ROS generation, a decrease in mitochondrial membrane potential, and a release of cytochrome c (cyt c) into the cytosol, leading to apoptosis [35,36,37,38] and inducing insulin-resistance/diabetes onset [39,40,41,42,43,44]. 

Mitochondria can use several mechanisms to maintain their physiological homeostasis [1,3,45]. Mitochondrial dynamic behaviour, i.e., the balance between fusion and fission processes, plays a key role in cell survival [46,47]. Impairments in mitochondrial dynamics have been associated with mitochondrial functional defects and many diseases, including nervous system impairment, endothelial dysfunction and insulin resistance/diabetes [48,49,50]. Dynamics machinery involves different proteins in complete fusion or fission processes. Regarding the fusion process, two different mitochondrial GTPases, mitofusin 1 (Mfn1) and mitofusin 2 (Mfn2) are involved in mitochondrial outer membrane fusion. On the other hand, mitochondrial inner membrane fusion is regulated by the GTPase Optical Atrophy 1 (Opa1), which is also involved in the mitochondrial cristae remodeling and the regulation of apoptosis [51]. Concerning the fission process, this mechanism is mainly mediated by the dynamin-related protein1 (DRP1), a cytosolic GTPase that interacts with a mitochondrial receptor protein known as fission protein 1 (Fis1), which forms a ring around mitochondria to induce fragmentation [46,52,53]. Fusion and fission events play a crucial role in cells to maintain lifespan and mitochondrial health. The exchange of materials between healthy mitochondria can occur through the fusion process [47,52], whereas fission processes are helpful to segregate and eliminate damaged mitochondria or a part of them. Moreover, organelles’ fragmentation ensures their distribution into the cells during cell division, and is helpful to guarantee ATP requirement [54]. These two-linked sets of opposed dynamic processes are critical to maintain mitochondrial redistribution and network and to allow the cell to respond to its ever-changing physiological conditions [46,47,55]. Experimental evidence has shown that mitochondrial dynamics could be regulated by several factors and/or diseases, such as inflammatory events, cancer, nutrients, and chemical stressors [56,57,58]. As far as we know, the effect of DDE on mitochondrial proteins involved in mitochondrial dynamic processes in the frame of adaptive or toxic response to different pesticide doses has not yet been analysed.

The main aim of the present study was to analyze the dose-dependent response to DDE exposition in terms of cell viability, mitochondrial protein fusion, and fission content in the human hepatocarcinoma cell line (HepG2). Scientists consider this cellular model suitable for in vitro studies on hepatic toxicology [59,60]. Firstly, we analysed the effect of a wide range of DDE doses on cell viability. Given DDE lipophilic property, we also analysed the effect of DDE in association with diverse dietary fatty acids (i.e., palmitate as a saturated fatty acid, oleate as a monounsaturated fatty acid, and omega 3 fatty acids mixture as polyunsaturated fatty acids) on cell viability and lipid accumulation. Then, we focused our attention on the dose-dependent response to DDE exposure in terms of the content of protein involved in mitochondrial dynamic behaviour, including the fusion proteins Mfn2 and Opa1 and the fission protein DRP1 to analyze a protein marker for outer mitochondrial membrane fusion, inner mitochondrial membrane fusion or mitochondrial fission, respectively. We also analysed the contents of a protein linked to mitochondrial function and dynamics, such as glucose-regulated protein 75 (GRP75), a molecular chaperone involved in mitochondria-ER communication [61], and the mitochondrial superoxide dismutase isoform 2 (SOD2) involved in the cellular response to oxidative stress [18]. The present work showed a dose-dependent decrease in cell viability associated with an adaptive mitochondrial response showing a pro-survival function in response to low DDE doses, but mitochondrial fragmentation and loss of cell viability in response to high DDE doses.

## 2. Materials and Methods

### 2.1. Cell Culture and Treatments

A HepG2 cell line was cultured in Minimum Essential Medium supplemented with 10% (*v*/*v*) fetal bovine serum, 1% (*v*/*v*) non-essential amino acids, 0.2 mM L-glutamine, 50 U/mL penicillin and 50 µg/mL streptomycin (Invitrogen SRL, Milan, Italy). Cells were maintained at 37 °C in a 5% CO_2_, 95% air-humidified atmosphere and passaged twice a week. To perform treatments with DDE, cells were seeded at the density of 4.0 × 104/cm^2^ and cultured for 24 h before stimulation. A stock solution of DDE was prepared by dissolving the pesticide in dimethyl-sulfoxide (DMSO). In each treatment, the final concentration of DMSO in the culture medium was 0.1%, which represents a non-toxic concentration for hepatocytes [62]. Cells were exposed to DDE for 24 h by using a wide range of concentrations (0.5 μM; 1 μM; 2.5μM; 5 µM; 10 µM; 25 µM; 50 µM and 100 µM). In a previous study on DDE effect on HepG2 cells [63], it has been reported that the range of DDE concentrations among 1 and 100 μM approximate to levels of p,p-DDE found in serum, breast milk, liver, or adipose tissues of people exposed to DDT [63,64,65].

A control group of cells (no-DDE treated group) was only treated with DMSO (0.1%). In the coincubation experiments with fatty acids, cells were co-treated for 24 h with the different doses of DDE and 250 µM of palmitate, oleate or eicosapentaenoic acid (EPA) + docosahexaenoic acid (DHA) 1:1 mixture.

### 2.2. Fatty Acid-BSA Conjunction Preparation

Oleate and Palmitate (Merck) 40 mM stock solutions were prepared by dissolving fatty acids in NaOH 0.1 M at 70 °C for 15 min under stirring. A 10% BSA fatty acid-free (Sigma-Aldrich, St. Louis, MO, USA) solution was dissolved at 55° in NaCl 0.9% and mixed to fatty acid solutions 4:1 *v*/*v*, respectively. The resulting 8 mM fatty acid stock solutions were filtered using a syringe equipped to filter (0.2 μm), aliquoted and stored at −20 °C. Concerning EPA and DHA (Merck, Kenilworth, NJ, USA), 40 mM stock solutions were prepared by dissolving fatty acids in methanol as indicated by the manufacturer and mixed 1:1 *v*/*v* to obtain a stock solution EPA + DHA. Then, fatty acids were co-incubated with BSA in a culture medium. The final fatty acids concentration used in the study (250 µM) was freshly prepared in a cell culture medium at the time of the treatments.

### 2.3. Cell Viability

The cytotoxicity was assessed by a 3-(4,5-dime-thylthiazol-2-yl)-2,5-diphenylte-trazolium (MTT) assay. This technique is based on the enzymatic conversion of MTT in mitochondria [66]. To assess cell viability in the presence of DDE, after DDE treatment for 24 h, 0.5 mg/mL of MTT was added to the cell medium and incubated for 1.5 h at 37 °C to allow MTT to be metabolized. The resulting formazan crystals were dissolved in 100 μL/well DMSO, as previously reported [67], and the absorbance of the resulting suspension was measured at 595 and 655 nm to subtract the background in each sample. Then, the percentage of cytotoxicity in treated cells was calculated according to the following equation: cytotoxicity (%) = (OD control group − OD treatment group)/OD control group × 100% [68]. MTT analysis was performed by using three different control groups of cells: non-treated cells (NT); DMSO-stimulated cells (used as the control group for DDE stimuli); BSA treated cells (used as the control group for fatty acids).

### 2.4. Western-Blot

Western blot analysis was performed as previously described [69]. The following primary antibodies were used: Mfn2 (sc-100560, 1:1000; Santa Cruz Biotechnology, Dallas, TX, USA); Opa1 (sc-30572, 1: 1000, Santa Cruz Biotechnology); DRP1 (sc-3298, 1:1000; Santa Cruz Biotechnology); SOD2 (PA5-30604, 1:1000, Thermo Scientific, Waltham, MA, USA); GRP75 (sc-13967, 1:2000; Santa Cruz Biotechnology). GAPDH (ab8245, 1:2000, Abcam, Cambridge, UK) was used as a loading control guide to normalize protein levels in samples. Appropriate secondary antibody anti-mouse, anti-rabbit (1:10,000; Bio-Rad Laboratories, Hercules, CA, USA) or anti-goat (1:5000, Santa Cruz Biotechnology) were used. 

### 2.5. Oil Red-O Staining

Oil red stain quantitative (spectrophotometric) and qualitative (microscopic) analyses were performed using a classic protocol to detect lipid droplets in cells [70]. Briefly, after treatment, cells were washed in PBS at 37 °C, fixed in 4% PFA for 45 min at room temperature, washed two-time with distilled water and stained as indicated by Oil-Red-O conventional protocols. To quantify oil-Red O accumulated in cells, the stain was extracted from lipid droplets by using isopropanol 100% and measured by using a 96 well plate reader at 490 nm. Quantitative and qualitative analyses were compared to non-stimulated cells.

### 2.6. Statistics

All data were expressed as median with range (*n* = 3) or interquartile of range (*n* > 3). Each experiment was performed in biological triplicate *(n* = 3) or more (*n* > 3). Technical replicates (1 to 3 replicates) of the same biological samples were also performed. One-way ANOVA test followed by Bonferroni’s post hoc test was used to test significant differences (GraphPad Software Inc., San Diego, CA, USA). Differences were considered statistically significant with a *p*-value < 0.05.

## 3. Results

### 3.1. Dose-Dependent Effect of DDE on Cell Viability

**Figure 1:**
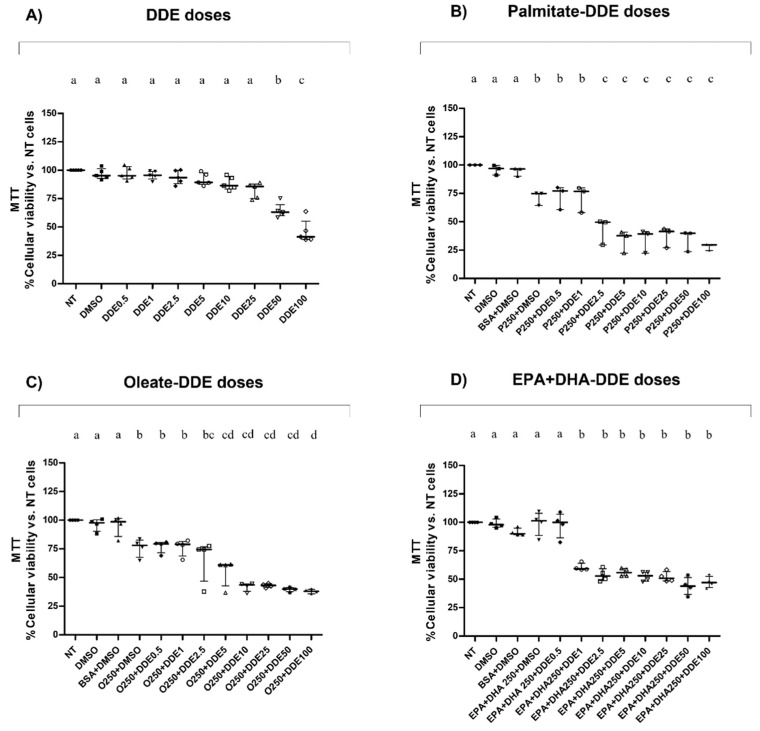
HepG2 cellular viability. Data were obtained stimulating cells with different doses of DDE (0.5 μM, 1 μM, 2.5μM, 5 µM, 10 µM,25 µM, 50 µM and 100 µM) (**A**) or with DDE in association with palmitate (250 µM) (**B**), oleate (250 µM) (**C**), or EPA + DHA (1:1) mixture (250 µM) (**D**). Results were graphically represented as median with range (**B**) or with interquartile or range (**A**,**C**,**D**) for 3 or 4 biological replicates. Statistical analyses were performed using GraphPad Prism software. One-way ANOVA analysis was followed by Bonferroni’s post-hoc test: different letters indicate statistically different values.

A dose-dependent decrease in cell viability was observed. No significant effect was found in the presence of low DDE doses up to 5 μM, whereas significant decreases in cell viability with values of 64% and 46%, compared to control cells (100%), were observed with high DDE doses (50 μM and 100 μM, respectively) (Figure 1A). Cell viability was also assessed in the presence of fatty acids. Compared to control, cells treated with 250 μM of palmitate or oleate reduced their viability values to 71% and 76%, respectively (Figure 1B,C), whereas cells treated with 250 μM EPA + DHA mixture did not show any changes in viability (Figure 1D). Moreover, cells cotreated with fatty acids and increasing DDE dose exhibited a higher dose-dependent reduction in viability compared to cells only treated with DDE. Cells co-treated with 250 μM of palmitate and different doses of DDE showed a significant dose-dependent decrease in cell viability starting with low doses, and showing values of 43%, 33% and 28% with 2.5 μM, 5 μM, and 100 μM DDE, respectively, compared to control cells (100%) (Figure 1B). A similar trend was observed with oleate coincubation, but with a lower degree of toxicity: cell viability was reduced to the values of 66%, 55% and 37% with 2.5 μM, 5 μM, and 100 μM DDE, respectively (Figure 1C). Dose-dependent decreases in cell viability values were less marked, mainly with high DDE doses when cells were cotreated with EPA + DHA mixture (54%, 56% and 47% values with 2.5, 5 and 100 μM DDE dose, respectively) (Figure 1D).

### 3.2. Dose-Dependent Effect of DDE on Lipid Depots in Cells Cotreated with Dietary Fatty Acids 

**Figure 2:**
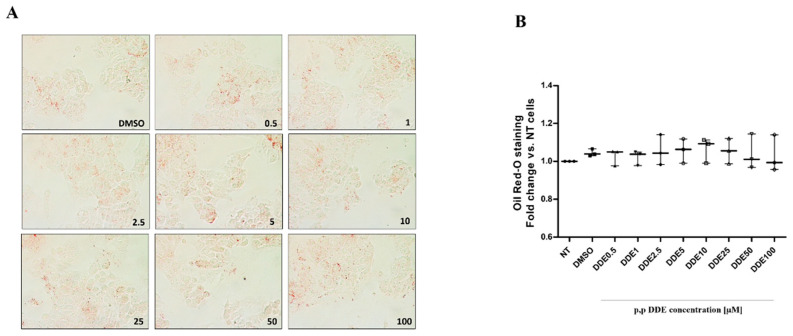
Total lipid content in response to different doses of DDE. Qualitative (**A**) and quantitative (**B**) analyses of total lipid content were performed using Oil-Red O staining. Panel (**A**) showed representative images of lipid droplets accumulated in cells after exposure to DMSO and DDE-doses. Original image magnification used: 40×. Panel (**B**) showed Oil-Red O quantification measured spectrophotometrically (490 nm). Data were graphically represented as median with range for 3 biological replicates.

**Figure 3:**
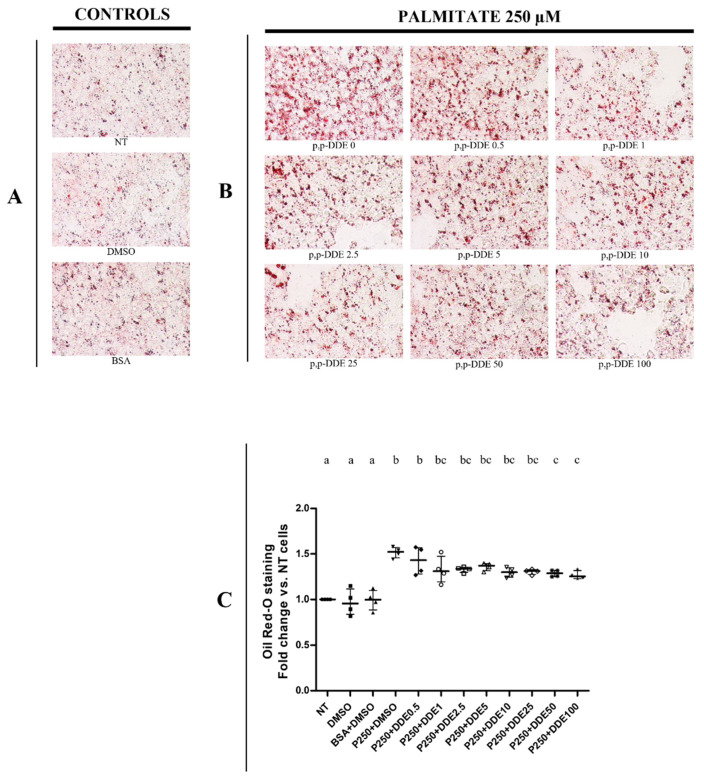
Total lipid content in response to different doses of DDE co-incubated with palmitate. (**A**) representative images of lipid depots in control cells (NT, DMSO, BSA); (**B**) representative images of cells treated with palmitate or with palmitate and increasing DDE doses. Original image magnification used: 40×. Quantitative analyses were obtained quantifying oil Red-O accumulation in cells. Oil-Red O quantifications were measured spectrophotometrically (490 nm). Data graphically represented as median with interquartile of range for 4 biological replicates (**C**). Three different control groups of cells were tested (non-treated cells, NT; DMSO and BSA) to evaluate the specific effect of DDE on fatty acid deposition. Statistical analyses were performed by using GraphPad Prism software. One-way ANOVA analysis followed by Bonferroni’s post-hoc test: different letters indicate statistically different values.

**Figure 4:**
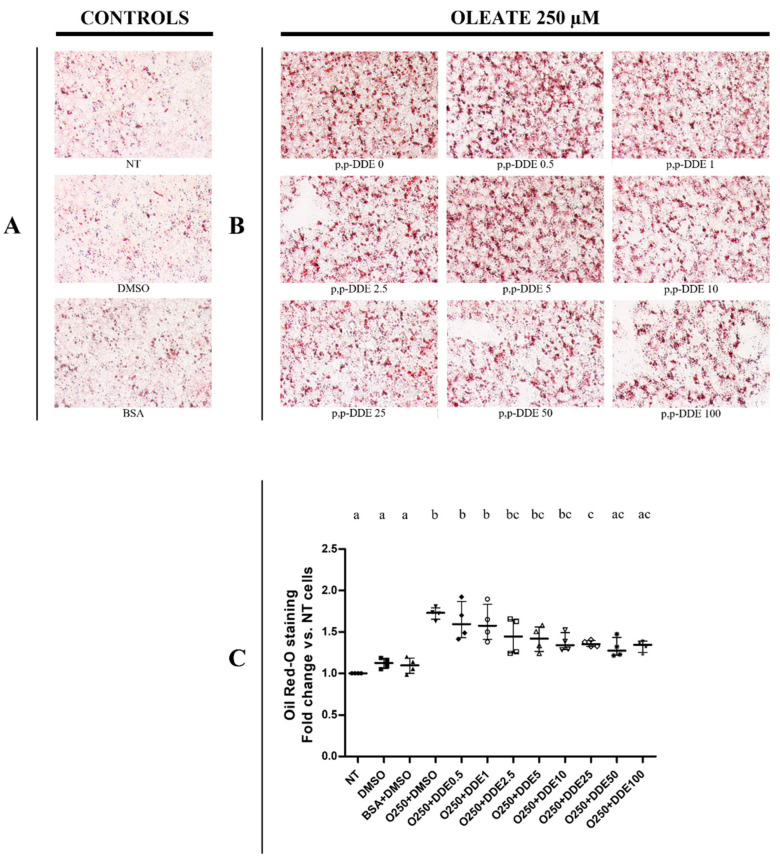
Total lipid content in response to different doses of DDE co-incubated with oleate. (**A**) representative images of lipid depots in control cells (NT, DMSO, BSA); (**B**) representative images of cells treated with oleate or with oleate and increasing DDE doses. Original image magnification used: 40×. Quantitative analyses were obtained quantifying oil Red-O accumulation in cells. Oil-Red O quantifications were measured spectrophotometrically (490 nm). Data graphically represented as median with interquartile of range for 4 biological replicates (**C**). Three different control groups of cells were tested (non-treated cells, NT; DMSO and BSA) to evaluate the specific effect of DDE on fatty acid deposition. Statistical analyses were performed by using GraphPad Prism software. One-way ANOVA analysis was followed by Bonferroni’s post-hoc test: different letters indicate statistically different values.

**Figure 5:**
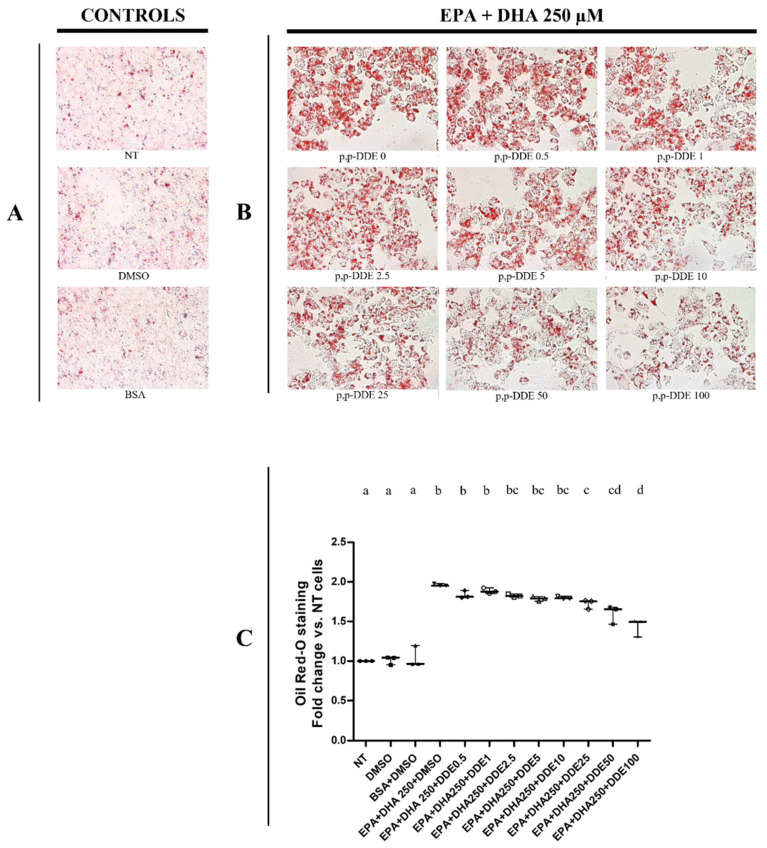
Total lipid content in response to different doses of DDE co-incubated with EPA/DHA mixture. (**A**) representative images of lipid depots in control cells (NT, DMSO, BSA); (**B**) representative images of cells treated with EPA + DHA or with EPA + DHA and increasing DDE doses. Original image magnification used: 40×. Quantitative analyses were obtained quantifying oil Red-O accumulation in cells. Oil-Red O quantifications were measured spectrophotometrically (490 nm). Data graphically represented as median with range for 3 biological replicates (**C**). Three different control groups of cells were tested (non-treated cells, NT; DMSO and BSA) to evaluate the specific effect of DDE on fatty acid deposition. Statistical analyses were performed by using GraphPad Prism software. One-way ANOVA analysis was followed by Bonferroni’s post-hoc test: different letters indicate statistically different values.

Qualitative and quantitative tests were performed using the oil-Red O method to detect total intracellular lipid accumulation in cells treated with DDE alone or associated with fatty acids. In a recent work, we analysed the time- and dose-dependent effect of palmitate or oleate on lipid accumulation and cell viability in HepG2 cells [71]. We showed that a significant changes in lipid depots and cell viability were observed in cells treated with 250 μM of fatty acids for 24 h, whereas lower doses did not induce significant effects [71]. Therefore, in the present work we decided to use the dose of 250 μM for fatty acids to analyze the possible effect of DDE on lipid depots induced by fat-treatments. In cells treated only with DDE, microscopic (Figure 2A) and spectrophotometric (Figure 2B) analyses did not show any changes in lipid accumulation between DDE-treated and DMSO-treated cells. As expected, lipid content significantly increased in the presence of fatty acids. Cells treated with 250 μM of palmitate (Figure 3), oleate (Figure 4) or EPA + DHA mixture (1:1) (Figure 5) increased lipid content by 52%, 72% and 98%, respectively, compared to control cells. Microscopy images showed that intracellular fat depots increased with the increasing degree of fatty acid unsaturation and that EPA + DHA treated cells exhibited the highest oil red staining. A slight progressive reduction in intracellular fat content in cells cotreated with fatty acids was observed in response to increasing DDE doses. Cells treated with 250 μM of palmitate and increasing doses of DDE, showed a dose-dependent decrease in fat depots, with an increase in fat content of 43% and 26% with 0.5 and 100 μM DDE, respectively, compared to control (Figure 3B,C), whereas palmitate alone induced a 52% increase. A similar trend was observed with oleate, with an increase of 63% and 33% in fat content with 0.5 and 100 μM DDE, respectively, compared to control (Figure 4B,C), whereas oleate alone induced a 72% increase. DDE dose-dependent increase in the intracellular fat depot was lower in the presence of EPA + DHA mixture with an increase of 83% and 43 % with 0.5 and 100 μM DDE, respectively, compared to control (Figure 5B,C), whereas EPA + DHA mixture alone induced a 98% increase.

### 3.3. Dose-Dependent Dffect of DDE on Mitochondrial Content of Proteins Involved in Fusion and Fission Processes

**Figure 6:**
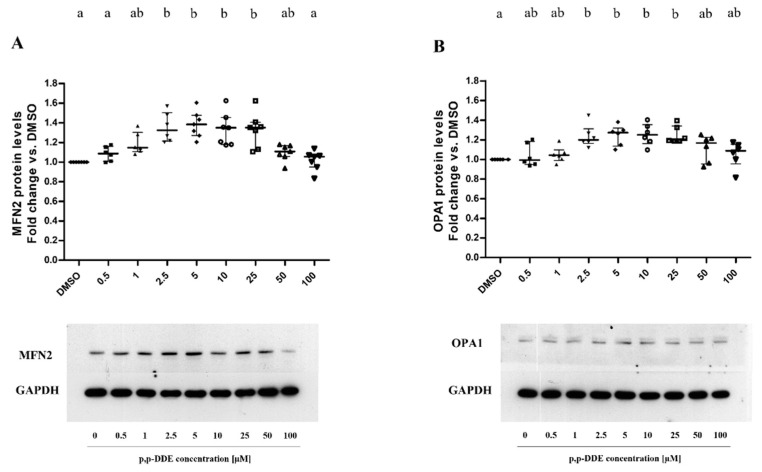
Mitochondrial fusion protein content. Mfn2 (**A**) and Opa1 **(B**) protein content were analyzed by western blotting. All data were presented as median with interquartile of range for 6 or 7 (Mfn2) and 5 (Opa1) biological replicates. Representative images of both Mfn2 (**A**) and Opa1 (**B**) (above) and the relative loading control for protein normalization GAPDH (below). Statistical analyses were performed using GraphPad Prism software. One-way ANOVA analysis followed by Bonferroni’s post-hoc test: different letters indicate statistically different values.

To evaluate the dose-dependent effect of DDE on mitochondrial dynamic machinery, western blot analysis was performed to measure cellular contents of proteins involved in mitochondrial fusion and fission processes, namely Mfn2 and Opa1, as a marker of outer and inner mitochondrial membrane, respectively, and DRP1 as a fission marker. Figure 6 shows that Mfn2 content significantly increased in response to DDE in a dose-dependent manner, reaching the highest increases (about +35%) in the 2.5–25 μM DDE dose range compared to control cells. However, Mfn2 contents progressively decreased with high DDE doses and reached the value of the control cells with the highest DDE dose (100 μM). Therefore, the dose-dependent response of Mfn2 content to DDE showed an inverted U-shape pattern (Figure 6A). In line with Mfn2 protein levels, Opa1 protein content described a similar inverted U-shape pattern (Figure 6B) and reached the highest increase (about +25%) in the 2.5–25 μM DDE range.

**Figure 7:**
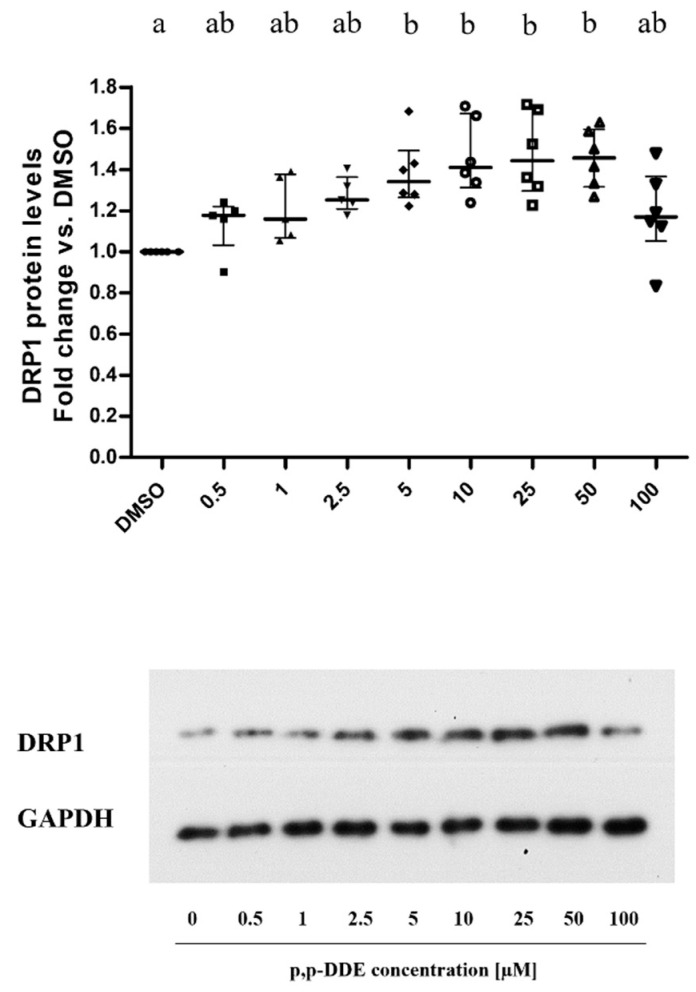
Mitochondrial fission DRP1 protein content. DRP1 protein levels were analyzed by western blotting. All data were presented as mean ± SEM for 5 or 6 biological replicates. The figure showed a representative image of DRP1 (above) and the relative loading control for protein normalization GAPDH (below). Statistical analyses were performed by GraphPad Prism software. One-way ANOVA analysis followed by Bonferroni’s post-hoc test: different letters indicate statistically different values.

As for fission marker DRP1, the relative protein content described a progressive-increase pattern in response to DDE concentrations up to the dose of 50 μM, with a significant increase (+45%) in the 5–50 μM range compared to control cells. On the other hand, the increase in DRP1 content was about +18% in cells treated with 100 μM of DDE (Figure 7). Therefore, the dose-response curve of DRP1 to increasing doses of DDE also showed an inverted U-shape pattern, with the highest point shifted towards higher doses than Mfn2 and Opa1 dose-response curves.

### 3.4. Dose-Dependent Effect of DDE on Mitochondrial Antioxidant Enzyme SOD2 and Mitochondria-ER Chaperone GRP75

**Figure 8:**
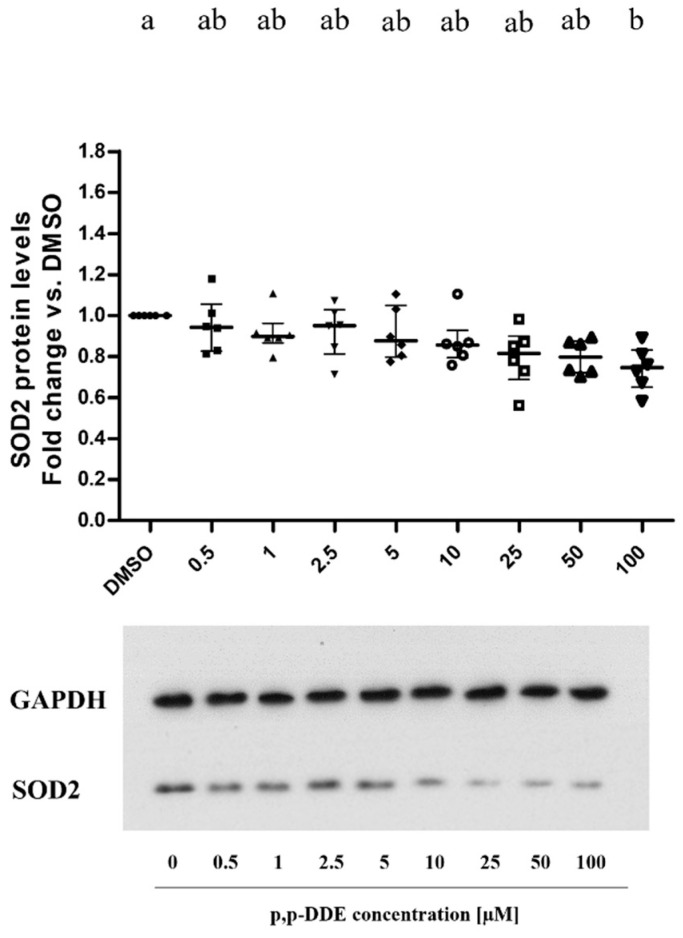
Mitochondrial antioxidant enzyme SOD2 protein levels. All data are presented as median with interquartile of range for 6 biological replicates. The figure showed representative images of SOD2 (below) and the relative loading control for protein normalization GAPDH (above). One-way ANOVA analysis followed by Bonferroni’s post-hoc test: different letters indicate statistically different values.

**Figure 9:**
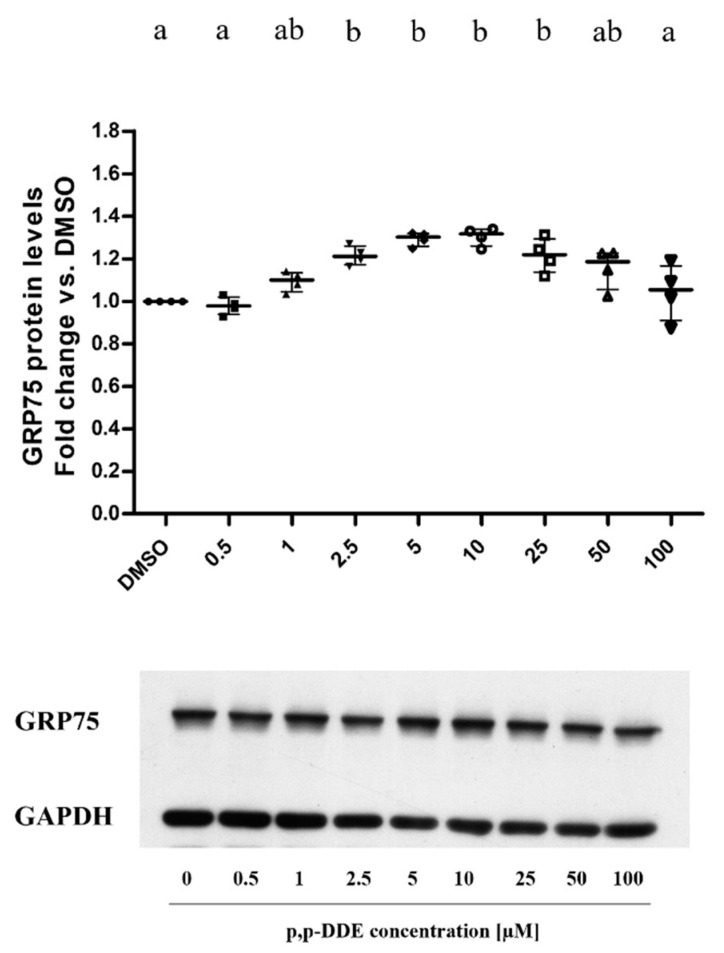
GRP75 protein levels. All data are presented as median with interquartile of range for 4 biological replicates. The figure shows representative images of GRP75 (above) and the relative loading control for protein normalization GAPDH (below). Statistical analyses were performed by using GraphPad Prism software. One-way ANOVA analysis followed by Bonferroni’s post-hoc test: different letters indicate statistically different values.

To study the effects of DDE on the mitochondrial antioxidant system, we analyzed the levels of mitochondrial enzyme SOD2 on total cell lysates. Results showed a progressive dose-dependent reduction in SOD2 protein content with a significant decrease (−25%) with the highest DDE dose (100 µM), compared to DMSO treated cells (Figure 8). Analyses of GRP75 levels in total cell lysates showed an inverted U-shape dose-response curve. A significant increase (+30%) in the protein content was found in the 2.5–25 µM DDE range compared to the levels measured in DMSO-treated cells. The highest DDE doses induced a decrease in GRP75 content compared to lower doses (Figure 9).

## 4. Discussion

Adverse effects of DDE, including cellular stress generation [17,72], apoptosis [73], and mitochondrial impairment [74], have been shown in both in vitro and in vivo studies. Present work aimed to further analyze the impact of DDE on cell viability and mitochondrial parameters in an in vitro hepatic cell model (HepG2 cells) in a dose-dependent approach. Indeed, we focused on the impact of increasing DDE doses in order to shed light on the adaptive or toxic outcomes in a dose-response approach. The novelty of the present work was in assessing cell viability under conditions of cotreatments with dietary fatty acids and evaluating the changes in mitochondrial dynamic proteins in association with cell viability maintenance or impairments in response to increasing DDE doses. 

Our study confirmed that DDE reduced cell viability in HepG2 cells in line with literature data in other cellular models [75]. Low DDE doses did not seem to have severe toxic effects, whereas high DDE doses (50 and 100 µM) elicited a significant reduction in cell viability. Given the lipophilic propriety of DDE and its ability to accumulate in lipid depots, we also analysed cell viability and intracellular fat depots under conditions of cotreatment with diverse dietary fatty acids, namely palmitate (as a saturated fatty acid), oleate (as a monounsaturated fatty acid) and EPA + DHA (1:1) mixture (as polyunsaturated fatty acids). Regarding the effect of fatty acids on cell viability, palmitate or oleate elicited a 25% or 20% reduction in cell viability, respectively. In contrast, the EPA + DHA mixture did not affect cell viability with the dose utilized in the present experimental work. Noteworthy, fatty acids induced an increase in DDE toxicity. Cells co-treated with fatty acids and DDE showed a significant reduction in cell viability even with low DDE doses, which did not elicit any significant decrease in cell viability in the absence of fatty acids. In cells cotreated with palmitate, 2.5 µM DDE dose dramatically decreased cell viability to the value of 43%, whereas a similar value (46%) was achieved with 100 µM DDE in the absence of fatty acids. In cells cotreated with oleate, a value of 44% cell viability was observed with 10 µM DDE dose. On the other hand, the EPA + DHA mixture elicited a decrease in cell viability to the value of 60% with 1 µM DDE dose and a decrease to the value of 47% with 100 µM DDE dose. It should be noted that cells cotreated with saturated fatty acid (palmitate) exhibited higher cell viability reduction with high DDE doses than cells cotreated with oleate or EPA + DHA. Indeed, the viability in cells cotreated with 100 µM DDE and unsaturated fatty acids decreased to a value (about 46%) similar to that observed in cells only treated with DDE, whereas in cells cotreated with palmitate, the reduction in cell viability was higher (28% compared to control cells). These data suggested that DDE associated with fatty acids was particularly detrimental for cell viability and that mainly saturated fatty acids could exacerbate environmental pollutants’ toxicity. In the presence of fatty acids, DDE reduced cell viability starting with low doses, suggesting the hypothesis that fatty acids could allow an easier DDE uptake into the cells leading to increased toxicity. Further experiments are needed to test this hypothesis. It should be noted that present results were obtained with the fatty acids dose of 250 μM that we previously showed to have a significant impact on cell viability compared to lower doses [71]. It could be of interest to test multiple doses of fatty acids to evaluate whether dose-dependent effect of DDE still remain when starting with low doses. 

It should also be noted that, in the presence of fatty acids, xenobiotics toxicity could be associated with lipo-toxicity with an additive adverse effect on cell survival [76]. Lipo-toxicity is the deleterious effect of lipid accumulation in non-adipose tissues and depends on the balance between fatty acids deposition and oxidation and the production of intermediate lipid metabolites, such as ceramides and diacylglycerols. We analysed intracellular lipid depots with a qualitative and quantitative test in the different experimental conditions. No changes in lipid depots were found in cells only treated with DDE, whereas intracellular lipid droplets increased in cells only treated with fatty acids. Quantitative analysis showed that lipid accumulation was higher in oleate than palmitate and further increased with EPA + DHA than oleate or palmitate. It should be noted that the highest lipid accumulation was observed in cells treated with unsaturated fatty acids that were less toxic toward cell viability compared to saturated fatty acids. This finding was in line with recent evidence, suggesting that the total triglycerides stored in liver cells were not the primary determinant of lipo-toxicity and specific lipid classes, such as ceramides and diacylglycerols, acted as damaging agents [77]. The hepatic accumulation of triglycerides in lipid droplets has been suggested to be protective rather than harmful in the progression from steatosis to non-alcoholic fatty liver disease [78,79]. 

Noteworthy, the coincubation of cells with fatty acids and increasing doses of DDE induced a progressive decrease in lipid accumulation compared to cells treated only with fatty acids. The reduction degree was similar for all fatty acids. A 20% decrease with low DDE doses and a 50% decrease with the highest DDE dose could be observed. Further experiments are needed to evaluate whether lower doses of fatty acids could induce a similar impact. However, present finding is in line with our previous results in an animal model of hepatic steatosis, where we showed that simultaneous treatment with DDE and high-fat diet elicited a significant reduction in hepatic lipid content associated with increased beta-oxidation rate and detoxification, compared to high-fat diet treatment in the absence of DDE [18]. It could be suggested that hepatocytes utilize fatty acids as metabolic substrates to sustain detoxification under the condition of partial exposure to xenobiotics.

Present findings in cells cotreated with increasing DDE doses and different types of dietary fatty acids suggested that it could be of interest to further investigate the interaction between DDE, as a lipophilic pollutant, and dietary fatty acids, given that they could be the main route of entry of DDE into the body. Further studies could shed light on the complex interactions among environmental pollutants and nutrients and suggest whether different dietary nutrients (such as saturated or unsaturated fatty acids) could potentiate the adverse effects of environmental pollutants or could be protective towards them.

A further goal of the present work was to analyze the dose-dependent response of mitochondrial markers to increasing doses of DDE in HepG2 cells. Mitochondria are cellular targets susceptible to chemicals, such as DDE [26]. Given that mitochondrial function is closely associated with mitochondrial morphology and cellular network [3], we analysed the main proteins involved in the mitochondrial dynamic machinery, namely proteins involved in fusion (Mfn2 and Opa1) and fission (DRP1) mechanisms. The balance between fusion and fission processes produces a mitochondrial network reorganization in response to various mitochondrial stressors and diseases [49]. Among others, our previous finding evidenced a possible role of mitochondrial dynamics in the control of cellular health and functionality in different tissues [55,80], suggesting the importance of studying these physiological mechanisms, mainly in the liver, that play a key role in lipid metabolism as well as in detoxification processes against external toxic agents [81]. We chose to analyze MFN2 because it has also been suggested that MFN2 is a protective target in the liver, as it controls the physiological balance between apoptosis and autophagy in liver failure [82]. As far as we know, this is the first time that mitochondrial proteins involved in mitochondrial dynamic behaviour are investigated in response to increasing doses of DDE in HepG2 cells. Our results demonstrated that both Mfn2 and Opa1 (fusion proteins) were modulated by DDE exposure in a dose-dependent manner, with a similar trend showing an inverted U-shape dose-response curve. Mfn2 and Opa1 contents progressively increased with increasing doses of DDE and reached the highest increase (about +30%) in a similar dose range (2.5–25 μM DDE). DRP1 fission protein content also increased with increasing DDE doses and reached the highest increase (+45 %) in the 10–50 μM doses range. It should be noted that both fusion and fission proteins analysed were increased by about 30% with the dose of 25 μM, whereas with the highest doses of 50 and 100 μM, a marked increase in protein content was still observed only for DRP1 (+45% and +18%). No significant changes were observed in Mfn2 and Opa1 content with the highest DDE dose (100 μM) compared to control cells. Our results showed that with low doses of DDE (up to 25 μM), a general increase in mitochondrial dynamic machinery was observed, suggesting an increase in mitochondrial mass through mitochondrial biogenesis, that could be useful to adapt cellular metabolism to face the increase in energy supply requirements for detoxication processes, and to counteract DDE toxicity. In line with this suggestion, our results showed that cell viability did not significantly decrease with low doses of DDE up to 25 μM. On the other hand, our results showed that the increase in DRP1 fission protein was still observed with the highest dose of DDE, in contrast to the increase in fusion proteins that was not found with the highest dose. This finding suggested that the balance between fusion and fission processes could be shifted towards mitochondrial fission with high DDE doses. This suggestion was supported by the increased DRP1/Mfn2 ratio of about 32% and 16% with 50 and 100 μM DDE doses, respectively, compared to control cells. It should be noted that cell viability was significantly reduced in cells treated with 50 and 100 μM DDE, suggesting a link between fission processes and reduction in cell viability. With the limitation that that fusion and fission protein contents should also be assessed in isolated mitochondria and that microscopy observations are needed to confirm mitochondrial dynamics and morphology changes, present results suggest the involvement of mitochondrial dynamics in the dose-dependent DDE effects on cell viability. Therefore, further analyses are needed to assess mitochondrial morphology network changes towards fission phenotype as a cellular response to high toxic DDE doses and confirm mitochondrial biogenesis as an adaptive cellular response to survive with low DDE doses.

In the present study, we also analysed SOD2 content as a marker of mitochondrial function and antioxidant system and GRP75 as a marker of mitochondria/endoplasmic reticulum interaction.

SOD2 represents one of the first mitochondrial-associated antioxidant defenses used by cells to quench superoxide anion produced by mitochondria [83]. It has been reported that reductions in SOD2 protein levels or functional defects in the enzymatical activity were associated with hepatic dysfunction and oxidative stress generation [84], a condition implicated in the etiology of many multifactorial chronic diseases [49,85,86,87,88]. In the present study, a decreasing trend in SOD2 content was observed in DDE-treated compared to DMSO cells, with a significant reduction with the highest DDE dose (about −25% vs DMSO). It has been suggested that the SOD2 enzyme ensures mitochondrial functional capacity, so its reduction could be associated with mitochondrial dysfunction [89,90]. Moreover, SOD2 has been suggested to improve the mitochondrial fusion process beyond its antioxidant activity [91]. Therefore, the decreased content of the SOD2 enzyme observed in our results could be associated with the shift toward fission processes in cells treated with the highest dose of DDE. 

We also analysed the content of intracellular chaperone GRP75, a protein involved in mitochondria/endoplasmic reticulum interaction [92]. This chaperone plays a crucial role in the generation of functional structures known as the mitochondrial associated membrane (MAMs). These contact points are essential to regulate calcium homeostasis, autophagy, lipid metabolism, mitochondrial morphology and cell survival [93,94]. It has been reported in the literature that GRP75 represents an essential protein involved in several cellular physiological processes. For example, chaperone overexpression prevents endoplasmic reticulum stress and apoptosis induced by glucose deprivation [95,96,97]. Moreover, Qiukay et al. [98] evidenced that GRP75 overexpression protected hepatic mitochondria from H_2_O_2_ and CCl4-induced oxidative damages, ameliorating ATP production and preserving cell viability. In our study, GRP75 protein levels showed a similar inverted U-shape dose-response curve as observed for Mfn2 and Opa1 levels, with the highest significant increase (about +30%) with 5 and 10 µM DDE vs DMSO-treated cells. This result suggested that an additional adaptive phenomenon involving ER-mitochondrial contact point formation/communication could be associated with mitochondrial increases in dynamic protein in response to low DDE doses. This mechanism could be used by cells to regulate cell survival and mitochondrial function in the presence of low DDE doses. On the one hand, the increase in mitochondrial dynamic machinery and GRP75 chaperone in response to low DDE doses could play a role in the mitochondrial network and mitochondria/endoplasmic reticulum interaction maintenance to maintain cell viability. On the other hand, in cells treated with high DDE doses, decreased levels in mitochondrial fusion proteins, GRP75, and SOD2 content associated with the increase in DRP1 levels suggested mitochondrial fragmentation and dysfunction in response to high DDE doses, which could trigger the observed reduction in cell viability.

## 5. Conclusions

The present study added new scientific notions regarding adaptive/toxic phenomena induced by increasing dose of DDE in hepatic cell culture. Cellular adaptation to low doses of the environmental pollutant seemed to include maintaining mitochondrial network and mitochondria-endoplasmic reticulum interaction, which could play a pro-survival role. On the other hand, high doses of DDE induced a toxic effect that reduced cell viability by impairing mitochondrial fusion/fission balance and antioxidant defense. Present results are useful to clarify the mechanisms underlying the cell fate towards survival or death in response to increasing doses of environmental pollutants. Further studies are needed to shed further light on the metabolic events associated with our findings and highlight the different roles of cellular organelles and their adaptation to environmental pollutants.

## Data Availability

All supporting data have been included in this study and are available from the corresponding authors upon request.

## References

[1] Meyer J.N., Leung M.C., Rooney J.P., Sendoel A., Hengartner M.O., Kisby G.E., Bess A.S. (2013). Mitochondria as a target of environmental toxicants. Toxicol. Sci..

[2] Wallace D.C. (2013). A mitochondrial bioenergetic etiology of disease. J. Clin. Investig..

[3] Ni H.M., Williams J.A., Ding W.X. (2015). Mitochondrial dynamics and mitochondrial quality control. Redox Biol..

[4] McBride H.M., Neuspiel M., Wasiak S. (2006). Mitochondria: More than just a powerhouse. Curr. Biol..

[5] Picard M., Wallace D.C., Burelle Y. (2016). The rise of mitochondria in medicine. Mitochondrion.

[6] Wallace K.B. (2017). Mitochondrial toxicity. Toxicology.

[7] Shaughnessy D.T., Worth L., Lawler C.P., McAllister K.A., Longley M.J., Copeland W.C. (2010). Meeting report: Identification of biomarkers for early detection of mitochondrial dysfunction. Mitochondrion.

[8] Moreira A.C., Machado N.G., Bernardo T.C., Sardao V.A., Oliveira P.J., Serra P.A. (2011). Mitochondria as a biosensor for drug-induced toxicity-is it really relevant?. Biosensors for Health, Environment and Biosecurity.

[9] Naranmandura H., Xu S., Sawata T., Hao W.H., Liu H., Bu N., Ogra Y., Lou Y.J., Suzuki N. (2010). Mitochondria are the main target organelle for trivalent monomethylarsonous acid (MMA(III))-induced cytotoxicity. Chem. Res Toxicol..

[10] Knecht A.L., Goodale B.C., Truong L., Simonich M.T., Swanson A.J., Matzke M.M., Anderson K.A., Waters K.M., Tanguay R.L. (2013). Comparative developmental toxicity of environmentally relevant oxygenated PAHs. Toxicol. Appl. Pharmacol..

[11] Bestman J.E., Stackley K.D., Rahn J.J., Williamson T.J., Chan S.S. (2015). The cellular and molecular progression of mitochondrial dysfunction induced by 2,4-dinitrophenol in developing zebrafish embryos. Differentiation.

[12] Brunst K.J., Baccarelli A.A., Wright R.J. (2015). Integrating mitochondriomics in children’s environmental health. J. Appl. Toxicol..

[13] Caito S.W., Aschner M. (2015). Mitochondrial redox dysfunction and environmental exposures. Antioxid. Redox Signal..

[14] Kang D., Hamasaki N. (2002). Maintenance of mitochondrial DNA integrity: Repair and degradation. Curr. Genet..

[15] Venkatraman A., Landar A., Davis A.J., Chamlee L., Sanderson T., Kim H., Page G., Pompilius M., Ballinger S., Darley-Usmar V. (2004). Modification of the mitochondrial proteome in response to the stress of ethanol-dependent hepatotoxicity. J. Biol. Chem..

[16] Migliaccio V., Sica R., Scudiero R., Simoniello P., Putti R., Lionetti L. (2019). Physiological adaptation to simultaneous chronic exposure to high-fat diet and dichlorodipheniletylhene (DDE) in Wistar rat testis. Cells.

[17] Migliaccio V., Lionetti L., Putti R., Sica R., Scudiero R. (2019). Combined effects of DDE and hyperlipidic diet on metallothionein expression and synthesis in rat tissues. Environ. Toxicol..

[18] Migliaccio V., Scudiero R., Sica R., Lionetti L., Putti R. (2019). Oxidative stress and mitochondrial uncoupling protein 2 expression in hepatic steatosis induced by exposure to xenobiotic DDE and high fat diet in male Wistar rats. PLoS ONE.

[19] Nadal A., Quesada I., Tudurí E., Nogueiras R., Alonso-Magdalena P. (2017). Endocrine-disrupting chemicals and the regulation of energy balance. Nat. Rev. Endocrinol..

[20] Elmore S.E., La Merrill M.A. (2019). Oxidative phosphorylation impairment by DDT and DDE. Front. Endocrinol..

[21] Harley K.G., Marks A.R., Bradman A., Barr D.B., Eskenazi B. (2008). DDT exposure, work in agriculture, and time to pregnancy among farmworkers in California. J. Occup. Environ. Med..

[22] Malusá E., Tartanus M., Danelski W., Miszczak A., Szustakowska E., Kicińska J., Furmanczyk E.M. (2020). Monitoring of DDT in agricultural soils under organic farming in Poland and the risk of crop contamination. Environ. Manag..

[23] van den Berg H., Manuweera G., Konradsen F. (2017). Global trends in the production and use of DDT for control of malaria and other vector-borne diseases. Malar. J..

[24] Schecter A., Colacino J., Haffner D., Patel K., Opel M., Papke O., Birnbaum L. (2010). Perfluorinated compounds, polychlorinated biphenyls, and organochlorine pesticide contamination in composite food samples from Dallas, Texas, USA. Environ. Health Perspect..

[25] Kidd K.A., Bootsma H.A., Hesslein R.H., Muir D.C., Hecky R.E. (2001). Biomagnification of DDT through the benthic and pelagic food webs of Lake Malawi, East Africa: Importance of trophic level and carbon source. Environ. Sci. Technol..

[26] Mota P.C., Cordeiro M., Pereira S.P., Oliveira P.J., Moreno A.J., Ramalho-Santos J. (2011). Differential effects of p,p’-DDE on testis and liver mitochondria: Implications for reproductive toxicology. Reprod. Toxicol..

[27] Arroyo-Salgado B., Olivero-Verbel J., Guerrero-Castilla A. (2016). Direct effect of p,p’-DDT on mice liver. Braz. J. Pharm. Sci..

[28] Liu Q., Wang Q., Xu C., Shao W., Zhang C., Liu H., Jiang Z., Gu A. (2017). Organochloride pesticides impaired mitochondrial function in hepatocytes and aggravated disorders of fatty acid metabolism. Sci. Rep..

[29] U.S. Environmental Protection Agency (USEPA) (2008). Health Effects Support Document for 1,1-Dichloro-2,2-bis(p-chlorophenyl)Ethylene (DDE).

[30] Holloway C.J., Brunner G., Schmidt F.W. (1981). The biochemistry of hepatic detoxification. Artificial Liver Support.

[31] Angrish M.M., Kaiser J.P., McQueen C.A., Chorley B.N. (2016). Tipping the balance: Hepatotoxicity and the four apical key events of hepatic steatosis. Toxicol. Sci..

[32] Cribb A.E., Peyrou M., Muruganandan S., Schneider L. (2005). The endoplasmic reticulum in xenobiotic toxicity. Drug Metab. Rev..

[33] Song L., Liu J., Jin X., Li Z., Zhao M., Liu W. (2014). P, p′-Dichlorodiphenyldichloroethylene induces colorectal adenocarcinoma cell proliferation through oxidative stress. PLoS ONE.

[34] Marouani N., Hallegue D., Sakly M., Benkhalifa M., Ben Rhouma K., Tebourbi O. (2017). P,p’-DDT induces testicular oxidative stress-induced apoptosis in adult rats. Reprod. Biol. Endocrinol..

[35] Shi Y.Q., Wang Y.P., Song Y., Li H.W., Liu C.J., Wu Z.G., Yang K.D. (2010). P,p’-DDE induces testicular apoptosis in prepubertal rats via the Fas/FasL pathway. Toxicol. Lett..

[36] Song Y., Liang X., Hu Y., Wang Y., Yu H., Yang K. (2008). P,p’-DDE induces mitochondria-mediated apoptosis of cultured rat Sertoli cells. Toxicology.

[37] Song Y., Shi Y., Yu H., Hu Y., Wang Y., Yang K. (2011). P,p’-Dichlorodiphenoxydichloroethylene induced apoptosis of Sertoli cells through oxidative stress-mediated p38 MAPK and mitochondrial pathway. Toxicol. Lett..

[38] Quan C., Shi Y., Wang C., Wang C., Yang K. (2016). P,p’-DDE damages spermatogenesis via phospholipid hydroperoxide glutathione peroxidase depletion and mitochondria apoptosis pathway. Environ. Toxicol..

[39] Sivitz W.I. (2010). Mitochondrial dysfunction in obesity and diabetes. US Endocrinol..

[40] Kim K.S., Lee Y.M., Kim S.G., Lee I.K., Lee H.J., Kim J.H., Kim J., Moon H.B., Jacobs D.R., Lee D.H. (2014). Associations of organochlorine pesticides and polychlorinated biphenyls in visceral vs. subcutaneous adipose tissue with type 2 diabetes and insulin resistance. Chemosphere.

[41] Bhatti J.S., Bhatti G.K., Reddy P.H. (2017). Mitochondrial dysfunction and oxidative stress in metabolic disorders—A step towards mitochondria based therapeutic strategies. Biochim. Biophys. Acta Mol. Basis Dis..

[42] Ramalingam L., Menikdiwela K., LeMieux M., Dufour J.M., Kaur G., Kalupahana N., Moustaid-Moussa N. (2017). The renin angiotensin system, oxidative stress and mitochondrial function in obesity and insulin resistance. Biochim. Biophys Acta Mol. Basis Dis..

[43] Verma S.K., Garikipati V.N.S., Kishore R. (2017). Mitochondrial dysfunction and its impact on diabetic heart. Biochim. Biophys. Acta Mol. Basis Dis..

[44] Lefranc C., Friederich-Persson M., Palacios-Ramirez R., Nguyen Dinh Cat A. (2018). Mitochondrial oxidative stress in obesity: Role of the mineralocorticoid receptor. J. Endocrinol..

[45] Burgos-Aceves M.A., Cohen A., Paolella G., Lepretti M., Smith Y., Faggio C., Lionetti L. (2018). Modulation of mitochondrial functions by xenobiotic-induced microRNA: From environmental sentinel organisms to mammals. Sci. Total Environ..

[46] Westermann B. (2010). Mitochondrial fusion and fission in cell life and death. Nat. Rev. Mol. Cell Biol..

[47] van der Bliek A.M., Shen Q., Kawajiri S. (2013). Mechanisms of mitochondrial fission and fusion. Cold Spring Harb. Perspect. Biol..

[48] Shenouda S.M., Widlansky M.E., Chen K., Xu G., Holbrook M., Tabit C.E., Hamburg N.H., Frame A.A., Caiano T.L., Kluge M.A. (2011). Altered mitochondrial dynamics contributes to endothelial dysfunction in diabetes mellitus. Circulation.

[49] Suárez-Rivero J.M., Villanueva-Paz M., de la Cruz-Ojeda P., de la Mata M., Cotán D., Oropesa-Ávila M., de Lavera I., Álvarez-Córdoba M., Luzón-Hidalgo R., Sánchez-Alcázar J.A. (2016). Mitochondrial dynamics in mitochondrial diseases. Diseases.

[50] Schmukler E., Solomon S., Simonovitch S., Solomon S., Simonovitch S., Goldshmit Y., Wolfson E., Michaelson D.M., Pinkas-Kramarski R. (2020). Altered mitochondrial dynamics and function in APOE4-expressing astrocytes. Cell Death Dis..

[51] Patten D.A., Wong J., Khacho M., Soubannier V., Mailloux R.J., Pilon-Larose K., MacLaurin J.G., Park D.S., McBride H.M., Trinkle-Mulcahy L. (2014). OPA1-dependent cristae modulation is essential for cellular adaptation to metabolic demand. EMBO J..

[52] Twig G., Elorza A., Molina A.J., Mohamed H., Wikstrom J.D., Walzer G., Stiles L., Haigh S.E., Katz S., Las G. (2008). Fission and selective fusion govern mitochondrial segregation and elimination by autophagy. EMBO J..

[53] Otera H., Wang C., Cleland M.M., Setoguchi K., Yokota S., Youle R.J., Mihara K. (2010). Mff is an essential factor for mitochondrial recruitment of Drp1 during mitochondrial fission in mammalian cells. J. Cell Biol..

[54] Mishra P., Chan D.C. (2014). Mitochondrial dynamics and inheritance during cell division, development and disease. Nat. Rev. Mol. Cell Biol..

[55] Meyer J.N., Leuthner T.C., Luz A.L. (2017). Mitochondrial fusion, fission, and mitochondrial toxicity. Toxicology.

[56] Lionetti L., Mollica M.P., Donizzetti I., Gifuni G., Sica R., Pignalosa A., Cavaliere G., Gaita M., De Filippo C., Zorzano A. (2014). High-lard and high-fish-oil diets differ in their effects on function and dynamic behaviour of rat hepatic mitochondria. PLoS ONE.

[57] Eisner V., Picard M., Hajnóczky G. (2018). Mitochondrial dynamics in adaptive and maladaptive cellular stress responses. Nat. Cell Biol..

[58] Ježek J., Cooper K.F., Strich R. (2018). Reactive oxygen species and mitochondrial dynamics: The Yin and Yang of mitochondrial dysfunction and cancer progression. Antioxidants.

[59] Ferreira F.M., Madeira V.M., Moreno A.J. (1997). Interactions of 2,2-bis(p-chlorophenyl)-1,1-dichloroethylene with mitochondrial oxidative phosphorylation. Biochem. Pharmacol..

[60] Knasmüller S., Mersch-Sundermann V., Kevekordes S., Darroudi F., Huber W.W., Hoelzl C., Bichler J., Majer B.J. (2004). Use of human-derived liver cell lines for the detection of environmental and dietary genotoxicants; current state of knowledge. Toxicology.

[61] Tubbs E., Theurey P., Vial G., Bendridi N., Bravard A., Chauvin M.A., Ji-Cao J., Zoulim F., Bartosch B., Ovize M. (2014). Mitochondria-associated endoplasmic reticulum membrane (MAM) integrity is required for insulin signaling and is implicated in hepatic insulin resistance. Diabetes.

[62] Nikolaou N., Green C.J., Gunn P.J., Hodson L., Tomlinson J.W. (2016). Optimizing human hepatocyte models for metabolic phenotype and function: Effects of treatment with dimethyl sulfoxide (DMSO). Physiol. Rep..

[63] Shabbir A., DiStasio S., Zhao J., Cardozo C.P., Wolff M.S., Caplan A.J. (2005). Differential effects of the organochlorine pesticide DDT and its metabolite p,p’-DDE on p-glycoprotein activity and expression. Toxicol. Appl. Pharmacol..

[64] Chu S., Covaci A., Schepens P. (2003). Levels and chiral signatures of persistent organochlorine pollutants in human tissues from Belgium. Environ. Res..

[65] Chu S., Covaci A., Jacobs W., Haraguchi K., Schepens P. (2003). Distribution of methyl sulfone metabolites of polychlorinated biphenyls and p,p’-DDE in human tissues. Environ. Health Perspect..

[66] Mosmann T. (1983). Rapid colorimetric assay for cellular growth and survival: Application to proliferation and cytotoxicity assays. J. Immunol. Methods.

[67] Ozbek N., Bali E.B., Karasu C. (2015). Quercetin and hydroxytyrosol attenuates xanthine/xanthine oxidase-induced toxicity in H9c2 cardiomyocytes by regulation of oxidative stress and stress-sensitive signaling pathways. Gen Physiol. Biophys..

[68] Lepretti M., Paolella G., Giordano D., Marabotti A., Gay F., Capaldo A., Esposito C., Caputo I. (2015). 4-Nonylphenol reduces cell viability and induces apoptosis and ER-stress in a human epithelial intestinal cell line. Toxicol. Vitr..

[69] de Lange P., Cioffi F., Senese R., Moreno M., Lombardi A., Silvestri E., De Matteis R., Lionetti L., Mollica M.P., Goglia F. (2011). Nonthyrotoxic prevention of diet-induced insulin resistance by 3,5-diiodo-L-thyronine in rats. Diabetes.

[70] Dhami-Shah H., Vaidya R., Udipi S., Raghavan S., Abhijit S., Mohan V., Balasubramanyam M., Vaidya A. (2018). Picroside II attenuates fatty acid accumulation in HepG2 cells via modulation of fatty acid uptake and synthesis. Clin. Mol. Hepatol..

[71] de Sousa I.F., Migliaccio V., Lepretti M., Paolella G., Di Gregorio I., Caputo I., Ribeiro E.B., Lionetti L. (2021). Dose- and Time-Dependent Effects of Oleate on Mitochondrial Fusion/Fission Proteins and Cell Viability in HepG2 Cells: Comparison with Palmitate Effects. Int. J. Mol. Sci..

[72] Torres-Avilés N.A., Albores-García D., Luna A.L., Moreno-Galván M., Salgado-Bustamante M., Portales-Pérez D.P., Calderón-Aranda E.S. (2016). Exposure to p,p’-DDE induces morphological changes and activation of the PKCα-p38-C/EBPβ pathway in human promyelocytic HL-60 cells. Biomed. Res. Int..

[73] Shi Y.Q., Li H.W., Wang Y.P., Liu C.J., Yang K.D. (2013). P,p’-DDE induces apoptosis and mRNA expression of apoptosis-associated genes in testes of pubertal rats. Environ. Toxicol..

[74] Auger C., Alhasawi A., Contavadoo M., Appanna V.D. (2015). Dysfunctional mitochondrial bioenergetics and the pathogenesis of hepatic disorders. Front. Cell Dev. Biol..

[75] Shi Y., Song Y., Wang Y., Liang X., Hu Y., Guan X., Cheng J., Yang K. (2009). P,p’-DDE induces apoptosis of rat Sertoli cells via a FasL-dependent pathway. J. Biomed. Biotechnol..

[76] Engin A.B. (2017). What Is Lipotoxicity?. Adv. Exp. Med. Biol..

[77] Svegliati-Baron G., Pierantonelli I., Torquato P., Marinelli R., Ferrer C., Chatgilialoglu C., Bartolini D., Galli F. (2019). Lipidomic biomarkers and mechanisms of lipotoxicity in non-alcoholic fatty liver disease. Free Radic. Biol. Med..

[78] Yamaguchi K., Yang L., McCall S., Huang J., Yu X.X., Pandey S.K., Bhanot S., Monia B.P., Li Y.X., Diehl A.M. (2007). Inhibiting triglyceride synthesis improves hepatic steatosis but exacerbates liver damage and fibrosis in obese mice with nonalcoholic steatohepatitis. Hepatology.

[79] Alkhouri N., Dixon L.J., Feldstein A.E. (2009). Lipotoxicity in non alcoholic fatty liver disease: Not all lipids are created equal. Expert. Rev. Gastroenterol. Hepatol..

[80] Migliaccio V., Sica R., Di Gregorio I., Putti R., Lionetti L. (2019). High-fish oil and high-lard diets differently affect testicular antioxidant defence and mitochondrial fusion/fission balance in male Wistar rats: Potential protective effect of ω3 polyunsaturated fatty acids targeting mitochondria dynamics. Int. J. Mol. Sci..

[81] Migliaccio V., Di Gregorio I., Putti R., Lionetti L. (2019). Mitochondrial involvement in the adaptive response to chronic exposure to environmental pollutants and high-fat feeding in rat liver and testis. Cells.

[82] Joaquim M., Escobar-Henriques M. (2020). Role of Mitofusins and Mitophagy in Life or Death Decisions. Front. Cell Dev. Biol..

[83] Zelko I.N., Mariani T.J., Folz R.J. (2002). Superoxide dismutase multigene family: A comparison of the CuZn-SOD (SOD1), Mn-SOD (SOD2), and EC-SOD (SOD3) gene structures, evolution, and expression. Free Radic. Biol. Med..

[84] Liu Y., Qi W., Richardson A., Van Remmen H., Ikeno Y., Salmon A.B. (2013). Oxidative damage associated with obesity is prevented by overexpression of CuZn- or Mn-superoxide dismutase. Biochem. Biophys. Res. Commun..

[85] Sherer T.B., Betarbet R., Greenamyre J.T. (2002). Environment, mitochondria, and Parkinson’s disease. Neuroscientist.

[86] Wallace D.C. (2005). A mitochondrial paradigm of metabolic and degenerative diseases, aging, and cancer: A dawn for evolutionary medicine. Annu. Rev. Genet..

[87] Koshiba T. (2013). Mitochondrial-mediated antiviral immunity. Biochim. Biophys. Acta.

[88] West A.P. (2017). Mitochondrial dysfunction as a trigger of innate immune responses and inflammation. Toxicology.

[89] Paul A., Belton A., Nag S., Martin I., Grotewiel M.S., Duttaroy A. (2007). Reduced mitochondrial SOD displays mortality characteristics reminiscent of natural aging. Mech. Ageing Dev..

[90] Flynn J.M., Melov S. (2013). SOD2 in mitochondrial dysfunction and neurodegeneration. Free Radic. Biol. Med..

[91] Bhaskar S., Sheshadri P., Joseph J.P., Potdar C., Prasanna J., Kumar A. (2020). Mitochondrial superoxide dismutase specifies early neural commitment by modulating mitochondrial dynamics. iScience.

[92] Honrath B., Metz I., Bendridi N., Rieusset J., Culmsee C., Dolga A.M. (2017). Glucose-regulated protein 75 determines ER-mitochondrial coupling and sensitivity to oxidative stress in neuronal cells. Cell Death Discov..

[93] Filadi R., Theurey P., Pizzo P. (2017). The endoplasmic reticulum-mitochondria coupling in health and disease: Molecules, functions and significance. Cell Calcium.

[94] Lee S., Min K.T. (2008). The interface between ER and mitochondria: Molecular compositions and functions. Mol. Cells.

[95] Heal R.D., McGivan J.D. (1996). Induction of the stress protein Grp75 by amino acid deprivation in CHO cells does not involve an increase in Grp75 mRNA levels. Biochem. Soc. Trans..

[96] Ikesugi K., Mulhern M.L., Madson C.J., Hosoya K., Terasaki T., Kador P.F., Shinohara T. (2006). Induction of endoplasmic reticulum stress in retinal pericytes by glucose deprivation. Curr. Eye Res..

[97] Liu Y., Liu W., Song X.D., Zuo J. (2005). Effect of GRP75/mthsp70/PBP74/mortalin overexpression on intracellular ATP level, mitochondrial membrane potential and ROS accumulation following glucose deprivation in PC12 cells. Mol. Cell. Biochem..

[98] Qiukay E., Liu X., Liu Y., Liu W., Zuo J. (2013). Over-expression of GRP75 inhibits liver injury induced by oxidative damage. Acta Biochim. Biophys. Sin..

